# Supplementary material for: Splice-Junction-Based Mapping of Alternative Isoforms in the Human Proteome
Source: Cell Rep. Author manuscript; Available in PMC 2020 Jan 15. (PMC6961840; doi:10.1016/j.celrep.2019.11.026)
Supplement: 2 [file NIHMS1546469-supplement-2.zip › DF1/PXD006675/VentricularSeptum/VentricularSeptum_32_CYBA_VGWSLVSHLEAPEPTLWCLWVNQGR.pdf]

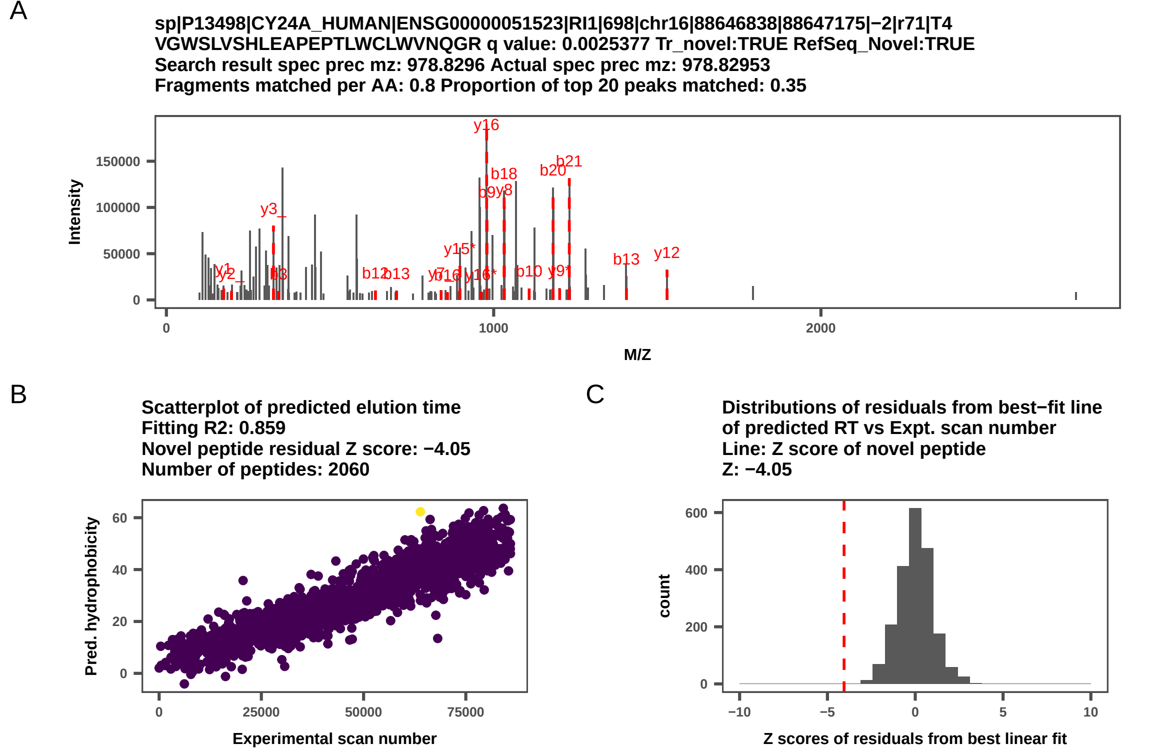
A
sp|P13498|CY24A_HUMAN|ENSG00000051523|RI1|698|chr16|88646838|88647175|−2|r71|T4
VGWSLVSHLEAPEPTLWCLWVNQGR q value: 0.0025377 Tr_novel:TRUE RefSeq_Novel:TRUE
Search result spec prec mz: 978.8296 Actual spec prec mz: 978.82953
Fragments matched per AA: 0.8 Proportion of top 20 peaks matched: 0.35
Intensity
150000
100000
50000
0
0
1000
2000
M/Z
y16
b18
b20
b21
b9y8
y3_
y15*
b13
y12
b12
b13
b10
y9*
b3
y1
y2_
B
Scatterplot of predicted elution time
Fitting R2: 0.859
Novel peptide residual Z score: −4.05
Number of peptides: 2060
Pred. hydrophobicity
60
40
20
0
0
25000
50000
75000
Experimental scan number
C
Distributions of residuals from best−fit line
of predicted RT vs Expt. scan number
Line: Z score of novel peptide
Z: −4.05
count
600
400
200
0
−10
−5
0
5
10
Z scores of residuals from best linear fit
